# HIV-1 Nef Uses a Conserved Pocket to Recruit the N-Terminal Cytoplasmic Tail of Serinc3

**DOI:** 10.3390/v18010005

**Published:** 2025-12-19

**Authors:** Mohammad Karimian Shamsabadi, Charlotte Stoneham, Amalia De Leon, Tony Fares, John Guatelli, Xiaofei Jia

**Affiliations:** 1 Department of Chemistry and Biochemistry, University of Massachusetts Dartmouth, Dartmouth, MA 02747, USA; 2 The Biomedical Engineering and Biotechnology Program, University of Massachusetts Dartmouth, Dartmouth, MA 02747, USA; 3 Department of Molecular Biology and Microbiology, Tufts University School of Medicine, Boston, MA 02111, USA; 4 Department of Medicine, University of California, San Diego, La Jolla, CA 92093, USA; 5 The VA San Diego Healthcare System, San Diego, CA 92161, USA; 6 Department of Biomedical Sciences, Florida State University College of Medicine, Tallahassee, FL 32306, USA

**Keywords:** HIV, Nef, Serinc, clathrin, downregulation

## Abstract

Human transmembrane proteins Serinc3 and Serinc5 are antiviral restriction factors that inhibit HIV-1 infectivity. In the absence of viral antagonism, Serinc3 and Serinc5 incorporate into the envelopes of nascent virions and inhibit the fusion of virions to the target cells. The HIV-1 virus counteracts the restriction of Serinc3 by downregulating it from the cell surface and thus excluding it from budding virions. This is orchestrated by the viral accessory protein Nef and involves hijacking of the clathrin adaptor protein complex 2 (AP2)-dependent endocytosis. The mechanistic details of Nef-mediated Serinc3 downregulation, however, have been enigmatic. In this work, we investigated and revealed the molecular determinants of Serinc3 modulation by Nef. Our results show that Nef recruits Serinc3 by binding to its N-terminal cytosolic tail. Furthermore, Nef residues important for Serinc3-binding in vitro, and for the exclusion of Serinc3 from virions, overlap with those required for Nef-mediated CD4 downregulation, suggesting great mechanistic similarities between the two functions of Nef. In addition to shedding light on the mechanism of Serinc3 antagonism, our work also highlights the conserved substrate-binding pocket of Nef as a molecular hotspot for inhibitor development and antiretroviral drug discovery.

## 1. Introduction

Serinc3 and Serinc5 proteins are members of the Serinc family, which additionally includes Serinc1, 2, and 4 [1]. Serinc proteins are multipass transmembrane proteins whose normal cellular functions are incompletely understood [2]. In 2015, Serinc3 and Serinc5 were identified, through two independent studies, as restriction factors of HIV-1 infection [3,4]. In the absence of viral antagonism, Serinc3 and Serinc5 are incorporated into the envelopes of the budding virions, leading to a reduction in the virions’ infectivity [3,4]. Specifically, their presence inhibits the fusion between the lipid membrane of the incoming virion and that of the target cell [3,4,5,6]. Not all strains of HIV-1 are subjected to restriction by Serinc3 and Serinc5; whether or to what extent the restriction occurs depends on the viral Env protein [5,7]. For the sensitive strains, the conformation of Env was found to be altered by the virion-incorporated Serinc3/5 [5,8,9,10], although this effect might not be caused by a direct Env–Serinc interaction [5]. While Serinc5 expression does not change the lipid composition of the viral or host cell membranes [11], Serinc3 and Serinc5 are lipid transporters that reduce the asymmetry of the lipid membrane [12]. Such lipid flipping activities of Serinc3/5 were shown to correlate with their restriction activities as well as with conformational changes in Env, providing a clue to the mechanistic basis of Serinc3/5’s antiviral activities [12].

The antiviral activities of Serinc3 and Serinc5 are antagonized by the HIV-1 accessory protein Nef [3,4]. Nef, a multifunctional peripheral membrane protein, is expressed early in the viral replication cycle and plays a key role in viral pathogenesis [13]. It modulates T cell activation through direct binding with cellular kinases, which presumably favors HIV-1 replication [14,15]. In addition, Nef downregulates immune-receptor molecules from the cell surface, enabling infected cells to evade both adaptive and innate immunity [13,16]. The best characterized Nef activities are downregulation of cell-surface CD4, which enables infected cells to evade antibody-dependent cellular cytotoxicity (ADCC) [17,18,19,20], and cell-surface downregulation of MHC-I, which hides infected cells from immune surveillance by cytotoxic T lymphocytes (CTLs) [21]. Nef executes the downregulation of surface CD4 by hijacking the clathrin AP2-dependent endocytosis [22,23], while downregulation of MHC-I takes place through Nef-mediated hijacking of clathrin AP1-dependent membrane trafficking [24,25,26,27].

**Figure 1:**
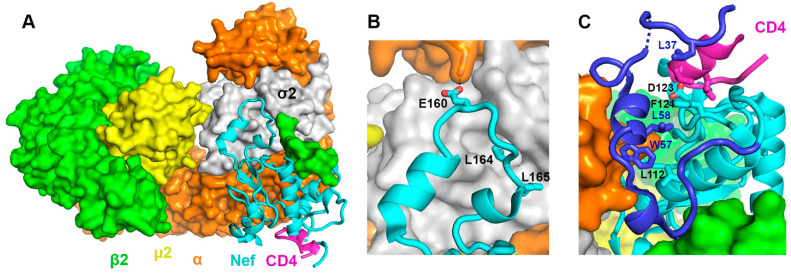
Mechanistic insights on Nef-mediated clathrin AP2-dependent CD4 downregulation gained by our previous crystal structure (PDB ID: 6URI) [32]. (**A**) Nef “connects” CD4 to the heterotetrameric clathrin AP2 complex. (**B**) The C-terminal loop of Nef binds extensively to the σ2 and α subunits of AP2 partly by mimicking the acidic dileucine motif. (**C**) Nef’s N-terminal loop (dark blue) adopts a unique conformation in the complex. Part of the loop forms a wall of the CD4-binding pocket; Leu37 within this loop makes direct contact with CD4. Phe121 and Asp123 on the core domain of Nef also interact with CD4. Nef residues Trp57 and Leu58 mediate the docking of the short helix into the hydrophobic pocket formed at the Nef–α interface.

Counteraction of Serinc3 and Serinc5 by Nef, like that of CD4, also occurs at the plasma membrane and similarly involves hijacking of clathrin AP2-dependent endocytosis [4,28,29]. AP2, the mediator of clathrin coat formation at the plasma membrane, is a tetrameric complex containing two large subunits (α and β2), one medium subunit (μ2), and one small subunit (σ2). AP2 recruits membrane cargos through binding to the sorting motifs located within the cytoplasmic domains of the cargo proteins. Two classes of motifs are commonly recognized by AP2 (as well as by other APs): the tyrosine-based motifs denoted as YxxΦ (Φ: a large hydrophobic residue; x: any amino acid) and the acidic dileucine motifs denoted as (E/D)xxxL(L/I) [30]. Nef hijacks AP2 partly through mimicking the acidic dileucine sorting motif: as revealed by high-resolution structures, the ExxxLL sequence located within the long and flexible C-terminal loop of Nef binds into a site on the σ2 and α subunits of AP2 dedicated to acidic dileucine motif binding (Figure 1A,B) [31,32]. The rest of Nef’s C-terminal loop also makes substantial interactions with the σ2 subunit of AP2 [31,32]. The extensive binding between Nef’s C-terminal loop and AP2, which has been observed with or without CD4 bound to Nef [31,32], is the foundation for the hijacking of the clathrin-mediated endocytosis by Nef.

While engaging AP2 through its C-terminal loop, Nef uses a different pocket to bind the cytoplasmic domain of CD4 (Figure 1A) [32]. Thus, Nef functions as a “connector” between AP2 and CD4. CD4-binding involves residues both from Nef’s rigid core (e.g., Phe121 and Asp123) and from its N-terminal loop (Leu37) (Figure 1C) [32]. Formation of this CD4-binding pocket depends on the Nef’s N-terminal loop adopting a unique conformation (Figure 1C). Here, two residues on the N-terminal loop—Trp57 and Leu58—dock into a shallow hydrophobic pocket on the Nef core, making direct contact with Leu112 and Phe121 [32]. Notably, mutations of these Nef residues, which are involved either in CD4-binding or in stabilizing the unique Nef conformation, indeed disrupted Nef-mediated surface downregulation of CD4 [22,23,32,33,34,35,36].

Downregulation of Serinc3 and Serinc5, which are similarly achieved through Nef-mediated hijacking of clathrin AP2-dependent endocytosis, prevents the incorporation of these innate restriction factors into virions and thus preserves virion-infectivity. Some mechanistic insights have so far been gained on the Serinc5-Nef interaction [2]. An intracellular loop of Serinc5, namely intracellular loop 4 or ICL4, is targeted by Nef for binding and downregulation [37]. Within ICL4, residues Ile350 and Leu353 are required [37]. On the Nef side, while certain determinants were found to be shared between the Serinc5 downregulation and the CD4 downregulation [11,38,39,40,41], some Nef residues were shown to be required for one downregulation but not the other, indicating distinction between the two mechanisms [42,43,44,45]. When it comes to the downregulation of Serinc3, however, the mechanism is largely enigmatic. It is not known which segment of Serinc3 is targeted by Nef, which residues or surface(s) of Nef are involved in recruiting Serinc3, or which conformation of Nef is responsible for this function. To fill this knowledge gap, here we investigated, and mapped, the molecular determinants of Nef-mediated Serinc3 antagonism. Our biochemical studies showed that Nef binds to the N-terminal cytoplasmic tail of Serinc3. Furthermore, Serinc3-binding was shown to involve the same Nef pocket, and the same Nef conformation, as CD4 downregulation does. The determinants in Nef-Serinc3 interaction, identified from in vitro studies, were subsequently validated in a cell-based assay, which measures the incorporation of Serinc3 into virions.

## 2. Materials and Methods

### 2.1. Accession ID

Proteins involved in this work, and their associated UniProt IDs, are listed below: HIV-1 Nef (Q90VU7); Serinc3 (Q13530); AP2 α subunit (P18484); AP2 σ2 subunit (P53680); AP2 β2 subunit (P63010); AP2 μ2 subunit (P96CW1).

### 2.2. Fusion Construct Design and Protein Expression and Purification

Genes encoding the Serinc3 intracellular loops (NTT: 1–39; ICL1: 120–131; ICL2: 181–204; ICL3: 259–267; and ICL4: 354–404) were each cloned into the pMAT9s vector, which allows the encoded protein to be expressed as a fusion to a N-terminal 6xHis-MBP tag that can be cleaved by the Mpro protease from SARS-CoV. *E. Coli* cells transformed with the plasmid were grown at 37 °C till OD_600_ reached 0.8. Protein expression was then induced with 0.1 mM IPTG and continued at 16 °C overnight. Harvested cells were lysed using sonication. The protein of interest was purified sequentially through an MBP affinity column, a HiTrap Q anion exchange column (GE Healthcare, Boston, MA, USA), and finally a Superdex 200 size exclusion column (GE Healthcare, Boston, MA, USA).

The construct of MBP-Nef was created by cloning the gene encoding 26–206 of NL4.3 Nef into the pMAT9s vector. Protein expression and purification follow the same steps as above.

The preparation of the α-Nef/σ2 protein used for the fluorescence polarization (FP) assay in Section 3.2 has been described previously [46]. Briefly, HIV-1 NL4.3 Nef (26–206) was fused to the C-terminus of α (1–398) subunit of AP2 via a 31-amino acid linker. This α-Nef fusion was then co-expressed with the σ2 subunit of AP2 (pCDFDuet-1 vector) in *E. coli* using conditions the same as the above. The α-Nef/σ2 complex was purified sequentially through a Ni-NTA affinity column, a HiTrap Q anion exchange column, and finally a Superdex 200 size exclusion column.

### 2.3. Binary Binding Test Using Size Exclusion Chromatography (SEC)

MBP-NTT and MBP-Nef (0.5 mg each) were mixed in a buffer solution (25 μM Tris, pH 8.0, 100 μM NaCl, 0.1 mM TCEP) with a final volume of 500 μL and incubated on ice for 1 h. The sample was then run through a Superdex 200 10/300 GL size exclusion column. MBP-NTT and MBP-Nef were also analyzed using SEC individually. The three elution profiles were overlaid to identify possible shifts. Other Serinc3 intracellular loop constructs were tested in the same fashion for possible binary binding with Nef.

### 2.4. Competition of TMR-Cyclic-CD4_CD_ by Unlabeled MBP-NTT/ICL in the FP Assay

The FP assay was developed previously, in which a tetramethylrhodamine (TMR)-labeled cyclized CD4 cytoplasmic domain (TMR-cyclic-CD4_CD_) binds to the α-Nef/σ2 protein to generate a high FP signal [46]. To test whether unlabeled MBP-NTT can displace TMR-cyclic-CD4_CD_ and thus decrease the FP signal, a stock solution of 120 µM unlabeled MBP-NTT was first prepared. The stock solution was then serial-diluted (2-fold each) 10 times. For making the final assay solutions, purified α-Nef/σ2 was buffer exchanged into the assay buffer (50 mM Tris, pH 8.0, 150 mM NaCl, 0.5 mM DTT, and 0.01% Triton X-100). The α-Nef/σ2 protein complex (25 µM) and TMR-cyclic-CD4_CD_ (200 nM) were first mixed in Corning 384-well black microplates (3820) and incubated at room temperature for 30 min. Then, MBP-NTT was added at different concentrations, the plate was incubated at room temperature for 2 h, and FP signals were then recorded using the EnVision plate reader (PerkinElmer, Shelton, CT, USA) with excitation at 535 nm and emission at 595 nm. All experiments were performed in triplicate. The FP data were fitted with non-linear regression using OriginLab. Statistical analysis was performed using Ordinary one-way ANOVA in GraphPad Prism 10. A *p*-value of <0.05 is considered statistically significant. Experiments using MBP-ICLs as competitors were performed the same way.

### 2.5. Labeling NTT with the TMR Fluorophore

The NTT(Cys16)-encoding gene was cloned into a pMAT9 vector. Expression and purification of MBP-NTT(Cys16) was carried out in the same way as MBP-NTT, described above. Conjugation of the TMR fluorophore onto NTT(Cys16) was performed by following a published protocol [47]. Briefly, 4 nmoles of purified MBP-NTT(Cys16) were buffer exchanged into TGED buffer (20 mM Tris, pH 7.9, 0.1 mM EDTA, 1 mM DTT, and 5% Glycerol). The proteins were then fully reduced by adding DTT (10 mM), followed by 2 h incubation at 4 °C. Saturated ammonium sulfate solution was then added to the sample to precipitate the protein. The sample was then centrifuged at 13,000 rpm for 5 min at 4 °C, and the supernatant was subsequently removed. The protein pellet was washed with buffer A (100 mM Na_2_PO_4_, pH 7.0, 200 mM NaCl, 1 mM EDTA) several times via centrifugation. The protein pellet was then dissolved in 100 µL of buffer A containing 20 nmoles of tetramethylrhodamine-5-maleimide and incubated at room temperature for 30 min. The excess reagents were removed through a desalting column, and labeled MBP-TMR-NTT was recovered afterwards. The MBP tag was subsequently removed through cleavage by the Mpro protease overnight, and the TMR-NTT was finally purified through a HiTrap Q anion exchange column.

### 2.6. Fluorescence Polarization Assay Using α-Nef/σ2 and TMR-NTT

The purified α(1–398)/σ2 (20 µM) and MBP-Nef (WT or mutant; 30 µM) were first mixed and incubated at room temperature for 30 min. Assays were carried out in Corning 384-well black microplates (3820). In each well, 200 nM TMR-NTT peptide was mixed with the proteins in a total volume of 15 μL. Incubation was performed for 1 or 2 h at room temperature with minimal exposure to light. Fluorescence polarization (FP) was then measured using the EnVision plate reader (PerkinElmer, Shelton, CT, USA) with excitation at 535 nm and emission at 595 nm. Measured FP values in triplicate were averaged and subsequently plotted using GraphPad Prism.

### 2.7. Plasmids and Mammalian Cells

Plasmid encoding pBJ5-Serinc3-HA was described previously [48]. The C-terminal HA epitope tag was removed, and an internal HA (YPYDVPDYA) was placed at position 314 (extracellular loop 4) using overlap PCR and restriction digestion at NotI and EcoRI sites in the pBJ5 plasmid backbone. Plasmids encoding Nef (from NL4-3; the wild-type referred to as pCINL below) and the indicated Nef mutants were based on pCI-neo (Promega, Madison, WI, USA) and previously described [28,32]. The proviral plasmids pNL4-3 and pNL4-3ΔNef were described previously [49,50,51]. The HEK293 cells were maintained in Dulbecco’s modified Eagle’s medium supplemented with 10% fetal bovine serum and penicillin/streptomycin.

### 2.8. Serinc3 Virion Incorporation and Immunoblotting

HEK293 cells seeded in 6-well plates were transfected with plasmid DNA encoding the HIV-1 molecular clone pNL4-3ΔNef (2.4 µg), pBJ5-Serinc3-HA (300 ng), pCINL (encoding Nef WT) or the indicated mutants (500 ng), and pCI-neo (empty plasmid) to reach a total of 4 µg DNA in each transfection mix, using Transporter 5 transfection reagent (PolySciences, Niles, IL, USA). The following day, culture supernates were clarified of cellular debris by centrifugation (1000× *g*), and the virions were partially purified from the supernates by ultracentrifugation of 1 mL at 23,500× *g* through 20% sucrose cushions. Virion-producer cells were harvested and pelleted by centrifugation (300× *g*). The viral and cell pellets were resuspended in Laemmli buffer containing 50 mM TCEP [Tris(2-carboxyethyl) phosphine; Sigma, Cream Ridge, NJ, USA]. To avoid boiling and the consequent aggregation of Serinc3, the samples were sonicated (Bioruptor, Diagenode, Denville, NJ, USA) before protein separation on 10% denaturing SDS-PAGE gels, transfer onto PVDF membranes, and immunoblotting with the antibodies indicated below. Immunoreactive bands were detected using the Western Clarity detection reagent (Bio-Rad, Hercules, CA, USA) and ChemiDoc imager system (Bio-Rad). The chemiluminescent signal was quantified using ImageLab v.5.1 software (Bio-Rad); the virion-associated Serinc3-HA signal was normalized to p24 and presented as a percentage signal of control (Serinc3-HA, no Nef).

Primary and secondary antibodies were prepared in antibody dilution buffer, consisting of 1% milk in phosphate-buffered saline (PBS) with 0.02% Tween 20 (PBST). The following antibodies were used for the detection of the proteins of interest: HA.11 (mouse; BioLegend, San Diego, CA, USA), GAPDH (glyceraldehyde-3-phosphate dehydrogenase; mouse; GeneTex, Irvine, CA, USA), HIV-1 p24 (mouse; Millipore, Burlington, MA, USA), and HIV-1-Nef (Rabbit; Abcam, Waltham, MA, USA). HRP-conjugated goat anti-mouse and donkey anti-rabbit secondary antibodies were from Bio-Rad.

## 3. Results

### 3.1. Nef Binds to the N-Terminal Cytoplasmic Tail of Serinc3 in Vitro

**Figure 2:**
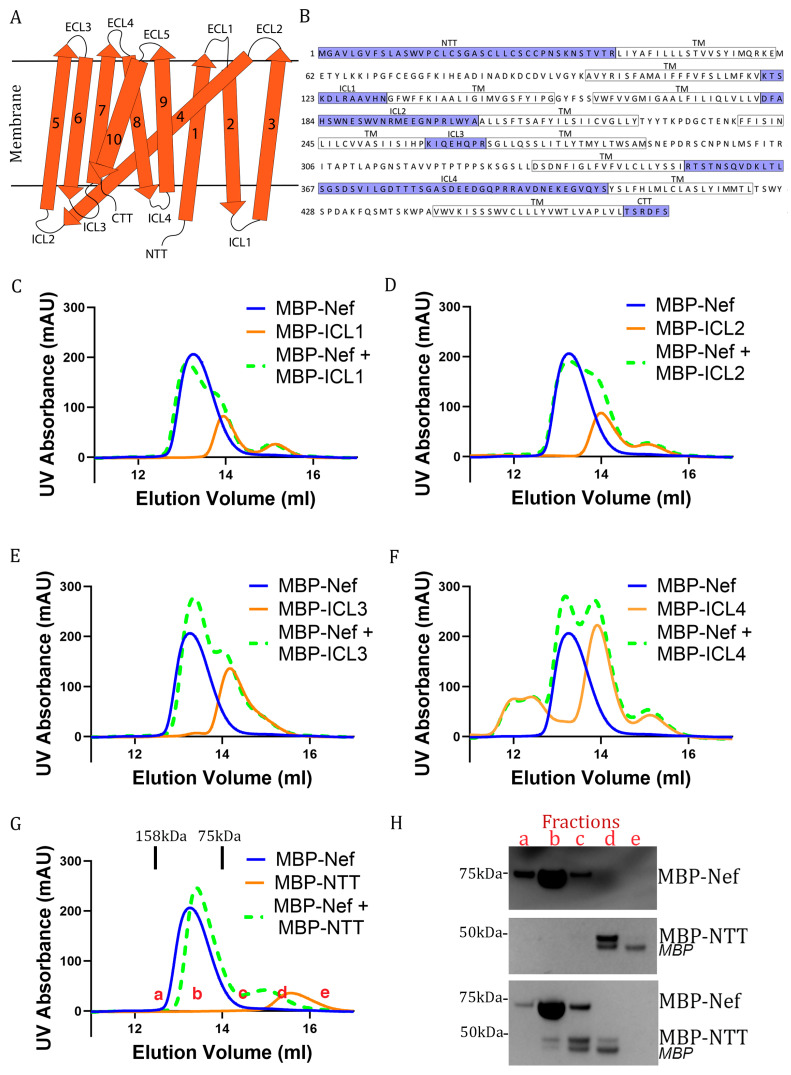
In vitro binding observed between Nef and the NTT of Serinc3. (**A**) Schematic of Serinc3 topology as a transmembrane protein with intracellular (ICLs) and extracellular loops (ECLs). Transmembrane helices are numbered, from N- to C-terminus, as 1–10. (**B**) Amino acid sequence of Serinc3 with intracellular segments highlighted. (**C**–**F**) SEC tests with MBP-ICLs showed that MBP-Nef does not bind to MBP-ICL1, MBP-ICL2, MBP-ICL3, or MBP-ICL4. (**G**) In the SEC test, the mixture of MBP-Nef and MBP-NTT exhibited an elution profile (green, dashed curve) that differs from the combination of the MBP-Nef elution profile (blue curve) and the MBP-NTT elution profile (orange curve). For reference, the elution positions of two protein standards with molecular weights of 158 kD and 75 kD, respectively, are marked. Notably, MBP-Nef alone (MW = 63.9 kD) was eluted between the two standards, consistent with its dimerization. (**H**) SDS-PAGE analysis of elution fractions (a–e) of SEC runs on MBP-Nef alone, MBP-NTT alone, and the mixture of the two. While the SEC tests for the ICLs have been performed only once, the SEC test for the NTT has been repeated three more times, and the binding signal was observed each time.

High-resolution structures solved by us and others have shown that, when downregulating host membrane proteins, Nef typically binds to a short cytoplasmic segment of its target [32,52,53,54]. Serinc3 is a multipass transmembrane protein and thus contains several intracellular segments: the N-terminal tail (NTT), intracellular loop 1 (ICL1), ICL2, ICL3, ICL4, and C-terminal tail (CTT) (Figure 2A,B) [12]. Two modes of binding are possible between Nef and Serinc3: (1) a single intracellular loop of Serinc3 is involved; (2) multiple intracellular loops are involved. In the related case of Nef-mediated Serinc5 downregulation, it has been shown that a single intracellular loop of Serinc5, namely ICL4, is mainly (if not solely) responsible. We hypothesized that Serinc3-Nef binding may similarly involve a single intracellular loop/segment of Serinc3. We also hypothesize that, even if more than one intracellular segment of Serinc3 is involved, a particular segment may be the main contributor and driver of the Serinc3-Nef interaction. To test our hypothesis, we decided to investigate each intracellular segment of Serinc3 individually for its possible binding with Nef. Among the Serinc3 intracellular segments, the CTT contains only six amino acids and is thus too short to be possibly targeted by Nef (Figure 2B). We therefore tested each of the remaining cytoplasmic segments of Serinc3—NTT, ICL1, ICL2, ICL3, and ICL4—for possible binding with Nef.

Each Serinc3 segment was fused to the C-terminus of the maltose-binding protein (MBP) and was subsequently expressed and purified. Nef was also expressed and purified as an MBP-Nef fusion. We then used a size exclusion chromatography (SEC)-based assay to detect possible binary binding between MBP-Nef and each of these MBP-Serinc3 fusions; here, binding between two proteins would be revealed by a shift in the elution volumes as bigger molecular species are eluted earlier in SEC. When the Serinc3 ICLs were tested in the SEC assay, no shift was exhibited when the elution profile of the MBP-Nef:MBP-ICL mixture and the elution profiles of individual proteins were compared, indicating that Nef does not bind to any of the Serinc3 ICLs (Figure 2C–F). Intriguingly, however, when the Serinc3 NTT was tested, the elution profile of the mixture of MBP-NTT and MBP-Nef displayed a significant shift compared to the elution profiles of individual proteins (Figure 2G). The shift in proteins’ elution positions was confirmed by SDS-PAGE analysis of eluted fractions: while MBP-NTT was not observed in fractions *b* and *c* when it was run on SEC alone (Figure 2H, middle gel), when mixed with MBP-Nef, MBP-NTT was observed in these two fractions (Figure 2H, bottom gel) consistent with its migration toward the higher MW region. These data showed that there is a binary interaction between Nef and the Serinc3 NTT.

We note that how the elution profiles of MBP-NTT and MBP-Nef shifted in the presence of each other was somewhat unexpected. In the presence of MBP-Nef, the elution of MBP-NTT shifted to the higher molecular weight (MW) region, which is consistent with complex formation between MBP-NTT and MBP-Nef (Figure 2G,H). On the other hand, however, in the presence of MBP-NTT, the elution of MBP-Nef shifted toward the lower MW region (Figure 2G,H). At first glance, this shift in MBP-Nef seemed counterintuitive because complex formation between two proteins typically results in both proteins being eluted in the higher MW region in SEC. However, careful analysis of this shift suggests to us that it may indicate an interesting aspect of the Nef-NTT binding. In the absence of MBP-NTT, MBP-Nef, which is 63.9 KDa in size, runs as a dimer on SEC (blue curve in Figure 2G; note its elution is between the reference positions of 75 KDa and 158 KDa). Since MBP-NTT (47.2 KDa) is smaller in MW in comparison to MBP-Nef, a 1:1 complex of MBP-Nef and MBP-NTT should be smaller in MW than the dimer of MBP-Nef. Thus, the MBP-NTT-induced shift in MBP-Nef from higher to lower MW region is consistent with the scenario that MBP-Nef is in the monomeric form when binding to MBP-NTT. Importantly, a similar scenario had been observed in the Nef-CD4 association: the conformation of Nef required for CD4-binding is incompatible with Nef dimerization [32].

### 3.2. Serinc3 NTT-Binding and CD4_CD_-Binding Involve the Same Conserved Pocket on Nef

**Figure 3:**
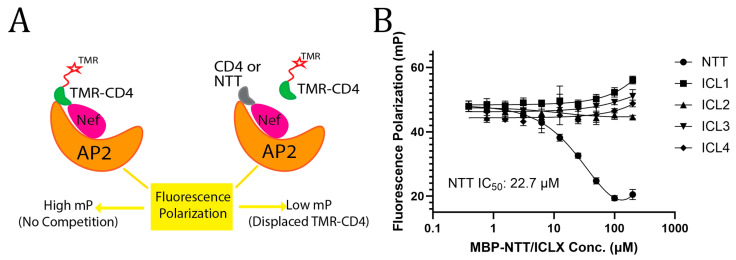
Serinc3 NTT and the CD4 cytoplasmic domain bind to the same multifunctional pocket of Nef. (**A**) Cartoon illustrating the design of the fluorescence polarization assay. (**B**) Addition of MBP-NTT led to a dose-dependent decrease in the FP signal (IC_50_: 22.7 µM) while no signal reduction was observed when any MBP-ICL was added. This experiment has been carried out once with three technical replicates. Analysis using one-way ANOVA indicated that the data of MBP-NTT competition are statistically significant (*p* = 0.0006).

Inspired by the similarity between Nef-CD4 binding and Nef-Serinc3 NTT binding as revealed by the SEC-based assays, we hypothesized that the Serinc3 NTT may bind into the conserved, multifunctional pocket of Nef—the same pocket that is responsible for binding CD4 in Nef-mediated, clathrin AP2-dependent downregulation and for binding MHC-I in Nef-mediated, clathrin AP1-dependent downregulation [32,52]. To test this, we used a previously established fluorescence polarization (FP) assay to investigate whether NTT and CD4_CD_ compete for binding to Nef (Figure 3A) [46]. This assay uses an engineered protein construct that functionally recapitulates the Nef-AP2 complex in vitro [46]. Specifically, the N-terminal half of the α subunit (residues 1–398) of AP2 was fused to the N-terminus of Nef through a flexible linker. This fusion chimera was then co-expressed and co-purified with the σ1 subunit of AP2. The resulting complex, named α-Nef/σ2, can associate with the fluorescent probe, tetramethylrhodamine-labeled cyclic CD4_CD_ (TMR-cyclic-CD4_CD_), to generate a significant FP signal [46]. Importantly, this FP signal is sensitive to competitive binding taking place at the CD4-binding pocket of Nef; addition of an unlabeled CD4_CD_ peptide caused a decrease in the FP signal in a dose-dependent manner [46]. Here, instead of unlabeled CD4_CD_, we added MBP-NTT to this assay, and the FP signal decreased in a dose-dependent manner (Figure 3B), which is consistent with a direct competition between MBP-NTT and the CD4-mimetic fluorescent probe. In contrast, none of the MBP-ICLs caused any decrease in the FP signal (Figure 3B). These results strongly indicate that the NTT-binding occurs, at least partially, at the conserved CD4-binding pocket of Nef. They also further confirmed that Nef specifically binds the NTT of Serinc3 but not any of its ICLs.

### 3.3. CD4_CD_- and Serinc3 NTT-Binding Share the Same Determinants in Nef and Should Involve the Same Conformation of Nef

**Figure 4:**
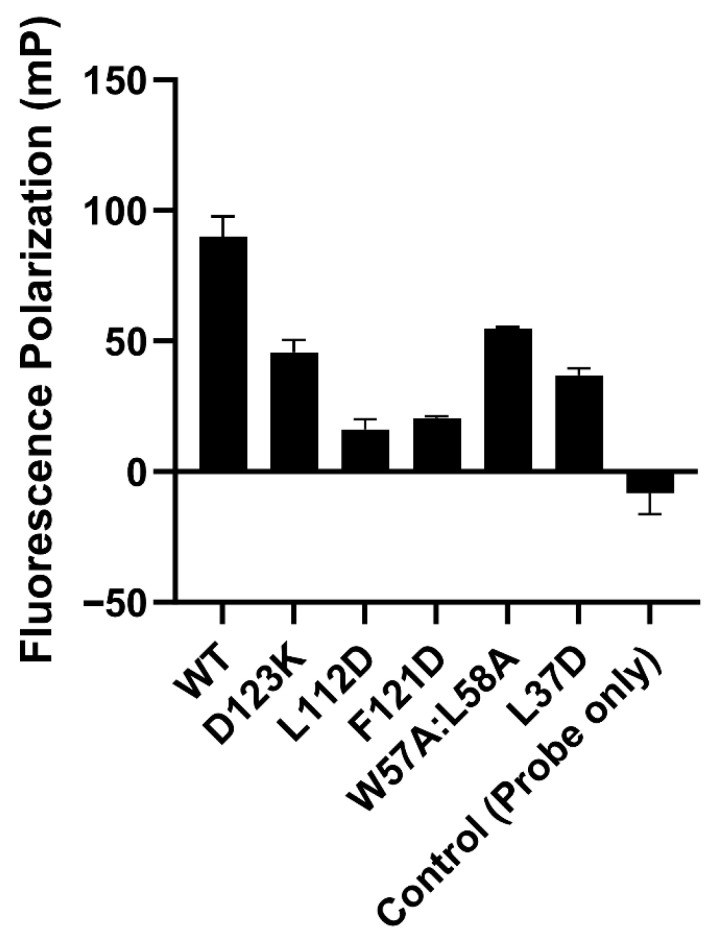
In vitro mutagenesis study revealed Nef residues important for Serinc3 NTT-binding. In the presence of α(1–398)/σ2 and the TMR-NTT probe, wild-type (WT) Nef produced a FP signal of ~90 mP in comparison to the control (probe only). All Nef mutations tested led to a reduction in FP signal to different degrees. Data are shown as mean and standard deviation of three technical replicates.

Our results discussed above pointed to some significant similarities between Serinc3 NTT and the CD4 cytoplasmic domain in binding Nef or the Nef-AP2 complex. Encouraged by these findings, we then tested, through in vitro mutagenesis, whether Nef residues important for CD4-binding are also required for binding Serinc3 NTT. Here, a modified FP assay was used to monitor the binding between NTT and the Nef-AP2 complex: a TMR-labeled NTT peptide was used as the fluorescent probe. When this probe was introduced to the complex containing wild-type Nef and the α-σ2 hemicomplex of AP2, a significant FP signal was observed compared to the background (Figure 4, WT vs. control), which is consistent with an association taking place between TMR-NTT and the Nef-containing complex.

We then investigated whether Nef mutations known to disrupt Nef-CD4 binding [32] could similarly disrupt the Nef-NTT association. The specific mutations tested are D123K, L112D, F121D, W57A:L58A, and L37D. Notably, not all Nef residues tested here contribute to CD4-binding through direct contact with CD4. While Nef residues Leu37, Phe121, and Asp123 make direct contacts with CD4, Trp57, Leu58, and Leu112 do not [32] (Figure 1C). To enable CD4 binding and downregulation, Trp57 and Leu58, which are located on a short helix within the Nef N-terminal loop, dock into a hydrophobic pocket on Nef core formed by Leu112, Phe121 and other residues (Figure 1C); this intramolecular association stabilizes a specific conformation of the Nef N-terminal loop and positions Leu37 and neighboring residues to bind CD4 [32]. Remarkably, when tested in our FP-based binding assay, each of the Nef mutants exhibited decreased FP signals, indicating that the binding to TMR-NTT was compromised by the mutation (Figure 4).

It is noteworthy that none of these mutations should affect the overall fold or stability of Nef. In fact, extensive mutagenesis studies had been carried out previously on Asp123 [3,26,32,33,34,35,55,56,57], Leu112 [32,34,35,38,39,57], Phe121 [32,35,38,39,57], and Trp57/Leu58 [22,23,58]. Furthermore, a previous study from Poe and colleagues showed that a quadruple mutant of Nef, I109D:L112D:Y115D:F121D, while exhibiting no activity in CD4 downregulation, remained competent in binding the SH3 domain of the Src-family kinase Hck [35]. Similarly, mutation of Asp123 of Nef did not affect the SH3-binding either [35]. Because the Nef-SH3 interaction requires Nef maintaining its three-dimensional structure [59,60], the study by Poe and colleagues thus indicated that the structural integrity of Nef should not be compromised by any of the three mutations: L112D, F121D, or D123K [35]. The L37D mutation has only been reported once previously [32]. However, Leu37 is within the flexible N-terminal loop of Nef; thus, the L37D mutation is unlikely to affect the three-dimensional fold of Nef. Finally, in the current study, during protein expression and purification, all five Nef mutants exhibited the same solution and chromatographic behavior as wild-type Nef, further suggesting that the overall fold of Nef is not affected by any of these mutations.

Overall, our results here revealed that Nef residues Asp123, Leu112, Phe121, Trp57, Leu58, and Leu37, which are highly conserved [32] and have been shown to be required in CD4 binding and/or downregulation by Nef [22,23,32,33,34,35,61], are also involved in the Nef-NTT association. Moreover, our results here suggest that the unique Nef conformation, which depends on the docking mediated by Trp57 and Leu 58 and is critical for CD4-binding (Figure 1C), should be similarly required for the Nef-NTT binding.

### 3.4. Virion Exclusion of Serinc3 Requires the Same Nef Residues Important for CD4 Downregulation

**Figure 5:**
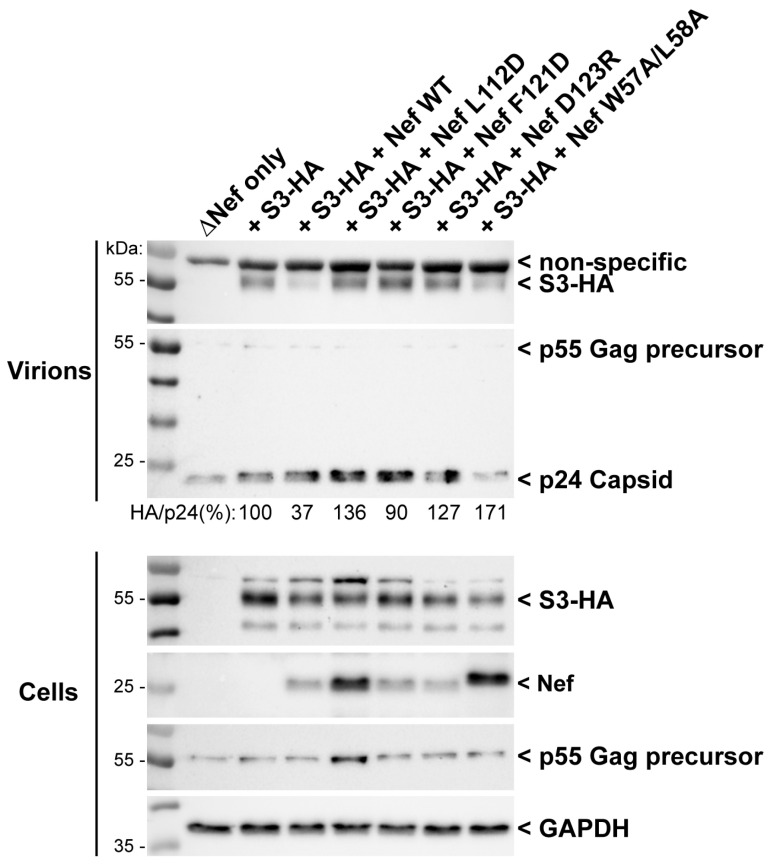
Nef residues important for CD4 downregulation are also required for excluding Serinc3 from virions. HIV-1 virions were produced in HEK293 cells co-transfected to express HIV-1 lacking an intact nef gene (ΔNef), Serinc3 (HA-tagged; S3-HA), and wild-type Nef (Nef WT) or the indicated Nef mutants, each from separate plasmids. Serinc3 (HA) was detected in virions partially purified from cell supernates by filtration and centrifugation through 20% sucrose cushions, and in virion producer cell lysates, by SDS PAGE and Western blot. The Western blots were also probed for Nef, GAPDH (a cellular protein used as a loading control), and p24/p55 Gag. The p55 Gag precursor is the predominant form of Gag in cells, while p24 predominates in virions following processing of p55 by the viral protease. A non-specific band detected by the anti-HA antibody is indicated; this was observed in the virion preparations even in the absence of S3-HA expression (far left sample lane; top blot). The Serinc3 (HA) and p24 bands were quantified in the virion Western blot images; the Serinc3 signal was normalized to p24 to adjust for variation in loading and expressed as a percentage of the control sample (HIV-1 virions produced with Serinc3 but without Nef). Western blot data are representative of two independent transfection experiments; Western blot images of the replicate experiment are shown in the supplemental data (Supplemental Figure S1).

As shown above, results from our in vitro tests converge and collectively suggest that Nef binds to the NTT of Serinc3 and likely downregulates Serinc3 through a mechanism similar to that of CD4 downregulation. We then aimed to validate these findings in human cells. We used a previously established assay to measure Nef-mediated exclusion of Serinc3 from virions by Western blot [48]. Nef-negative (ΔNef) HIV-1 virions were produced from HEK293 cells in the presence of Serinc3 containing an HA epitope tag, either with or without co-expression of wild-type Nef or Nef mutants. As expected, Serinc3 was detected in partially purified virions (Figure 5). The expression of wild-type Nef reduced the amount of Serinc3 in virions to 37% of that in the Nef-negative control. Mutations of residues within the CD4-binding pocket, F121D and D123R, which are known to disrupt CD4 binding and downregulation [32,33,34], restored the levels of Serinc3 in virions (Figure 5). These data confirm our observations in vitro and further support that the conserved pocket of Nef is involved in recruiting Serinc3 in cells and thus excluding Serinc3 from virions. We also tested the L112D and the W57A:L58A mutations, which, as discussed, should prevent Nef from adopting the distinct conformation critical for CD4 binding and downregulation. Consistent with our in vitro mutagenesis results, these mutations also inhibited Nef-mediated exclusion of Serinc3 from virions (Figure 5). Notably, we observed little or no degradation of total cellular levels of Serinc3 in the presence of Nef WT, despite that Nef reduced the incorporation of Serinc3 into virions. Endo-lysosomal degradation of Serinc5 by Nef has been reported, but this has not been evident in all studies, and moreover, might not apply to Serinc3 [4,39]. Virion-exclusion, as seen here, remains the best correlate of Nef-mediated Serinc antagonism.

## 4. Discussion

Previous work on Serinc5 showed that HIV-1 Nef targets the ICL4 of Serinc5 for downregulation and antagonism [37]. It has been subsequently speculated that Serinc3 downregulation may similarly involve Nef binding to the ICL4 of Serinc3 [12]. However, results from our in vitro assays—a SEC binding assay and two FP-based assays—converged in supporting that Nef binds directly to the N-terminal cytoplasmic tail of Serinc3 (Figure 2, Figure 3 and Figure 4) but not any of its ICLs (Figure 2 and Figure 3). A limitation of our study is that we tested each intracellular segment of Serinc3 separately, which should have prevented us from evaluating whether multiple segments of Serinc3 could be involved in Nef/AP2-binding simultaneously. We believe, however, that, even if multiple segments of Serinc3 are involved, the NTT of Serinc3 should still be a major contributor to the Nef-Serinc3 association. In support of this, we showed that mutations of Nef residues, which disrupted Nef-NTT association in vitro (Figure 4), also abolished Nef’s activity in excluding Serinc3 from budding virions (Figure 5).

Although Serinc5 and Serinc3 are similar both structurally and functionally [3,4,12,62], different segments of Serinc5 and Serinc3 are targeted by Nef for their respective downregulation: Nef antagonizes Serinc5 through binding to its ICL4, while the downregulation of Serinc3 involves Nef associating with the N-terminal tail of this host factor. This finding highlights the functional versatility of Nef. It also suggests that, despite that Serinc3 is less potent than Serinc5 in restricting HIV-1 [3,4], Serinc3 modulation is unlikely a “side-effect” of Serinc5 antagonism but is more likely a deliberate function of Nef shaped through evolution. A recent study revealed that Serinc3 is present in extracellular vesicles (EVs) and that its distribution within different populations of EVs is altered upon HIV-1 infection in a Nef-dependent manner [63]. Conceivably, the Nef-mediated incorporation of Serinc3 into EVs, which is unique to Serinc3 and not observed in Nef’s modulation of other cellular targets [63,64], may benefit HIV-1 in ways beyond merely restoring viral infectivity. It also suggests that, in addition to the plasma membrane, Nef may interact with Serinc3 in other cellular compartments (e.g., multivesicular bodies, a place known for the biogenesis of EVs) to modulate its transportation and/or cellular level.

Surface downregulation of the Serinc proteins, like that of CD4, occurs through Nef hijacking of clathrin AP2-mediated endocytosis [4,28,29]. As revealed by our previous crystal structure of Nef in complex with CD4_CD_ and AP2, while Nef uses mainly its C-terminal loop to bind AP2, CD4-binding involves a separate set of Nef residues and requires Nef to adopt a specific conformation [32]. Our work here shows that the same binding pocket, and the same conformation of Nef, should be involved in Serinc3 modulation: mutation known to disrupt the CD4-Nef interaction (D123K), mutations known to prevent Nef from adopting the specific conformation for CD4 binding (W57A:L58A and L112D), and mutation affecting both (F121D) all disrupted Nef-Serinc3 NTT binding (Figure 4). Consistently, the same mutations also disrupted Nef-mediated virion exclusion of Serinc3 (Figure 5) [32].

The downregulation of Serinc5 also shares many of the same determinants in Nef. Mutations involving Trp57, Leu112, and Phe121 in Nef each impaired Serinc5 downregulation [11,38]; the same effect was also observed when the Nef N-terminal residues 12–39 were truncated [40]. While these and other findings point to great similarities between the modulation of Serinc5, Serinc3, and CD4, differences between the determinants of these Nef functions have also been reported. Studies, including those on Nef polymorphisms, showed that Serinc5 downregulation and CD4 downregulation are to some extent genetically separable—some Nef mutations differentially affect and thus potentially uncouple these two functions [42,43,44,45]. Similarly, Nef proteins from different subtypes of HIV-1 [65], as well as from different patients [66], vary in their abilities to antagonize Serinc5 and Serinc3, suggesting that these might also be genetically separable functions of Nef. On the other hand, the CD4 cytoplasmic domain, the Serinc3 N-terminal tail, and the Serinc5 ICL4 do not share sequence similarities with one another. Complete elucidation of the mechanisms of Nef-mediated downregulation of Serinc3/5 will require high-resolution structures of the corresponding complexes; we aim to solve such structures in the future.

Finally, the revelation that Serinc3-binding occurs at the conserved Nef pocket—the same site involved in recruiting CD4 and MHC-I [32,52]—further underscores the significance of this pocket in drug discovery against Nef. Small molecules that bind avidly to this site should be able to rescue several cellular targets of Nef at once, which may revitalize multiple adaptive and innate immune mechanisms to combat HIV-1 infection. Such therapeutics, if developed successfully, could be of great impact as they are directly applicable to HIV-1 cure strategies.

## Data Availability

The original contributions presented in this study are included in the article/Supplementary Material. Further inquiries can be directed to the corresponding author.

## Supplementary Materials

The following supporting information can be downloaded at: https://www.mdpi.com/article/10.3390/v18010005/s1, Figure S1: Replicate experiment of that of Figure 5.
