# Supplementary figures and images for: HIV-1 Nef Uses a Conserved Pocket to Recruit the N-Terminal Cytoplasmic Tail of Serinc3
Source: Viruses. 2025 Dec 19;18(1):5. doi: 10.3390/v18010005 (PMC12846431; doi:10.3390/v18010005)

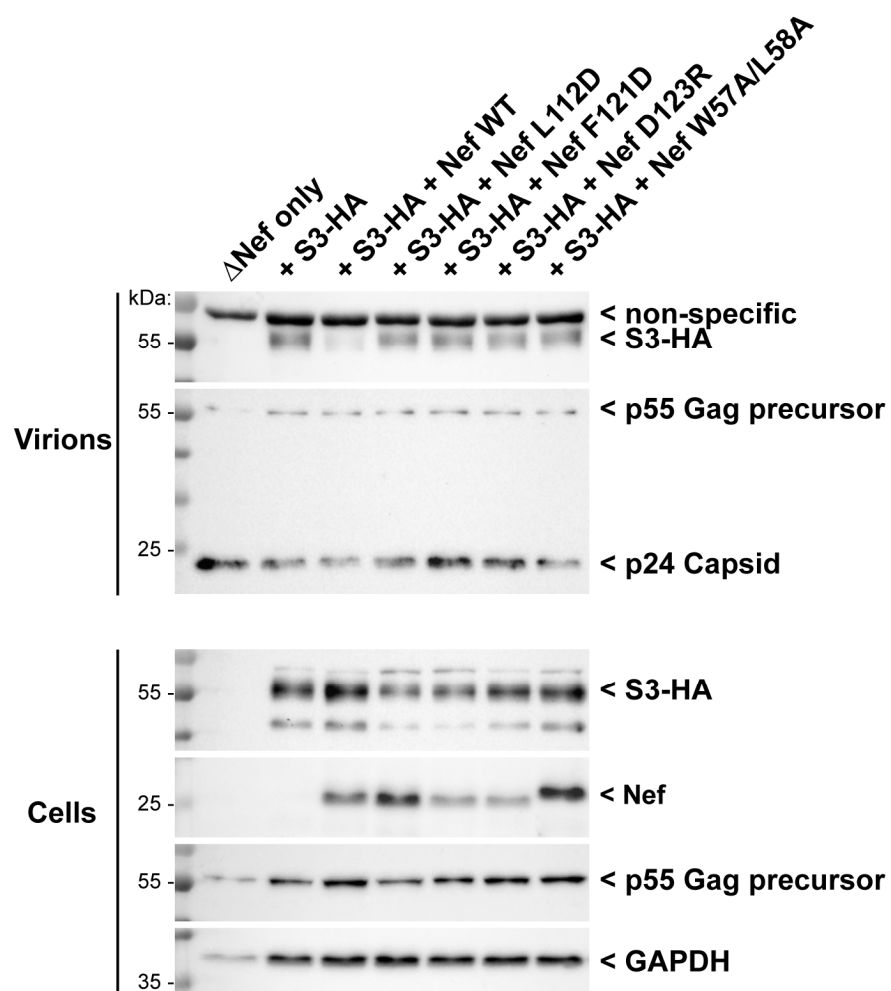

**Supplemental Figure 1.** Replicate experiment of that of Figure 5.

Supplement: Supplementary file 1 [file viruses-18-00005-s001.zip › viruses-3974790-supplementary.pdf]
